# Batch Reactor vs. Microreactor System for Efficient AuNP Deposition on Activated Carbon Fibers

**DOI:** 10.3390/ma14216598

**Published:** 2021-11-02

**Authors:** Magdalena Luty-Błocho, Marek Wojnicki, Tomasz Tokarski, Volker Hessel, Krzysztof Fitzner

**Affiliations:** 1 Faculty of Non-Ferrous Metals, AGH University of Science and Technology, al. A. Mickiewicza 30, 30-059 Krakow, Poland; marekw@agh.edu.pl (M.W.); fitzner@agh.edu.pl (K.F.); 2 Academic Centre for Materials and Nanotechnology, AGH University of Science and Technology, al. A. Mickiewicza 30, 30-059 Kraków, Poland; tokarski@agh.edu.pl; 3 School of Chemical Engineering and Advanced Materials, University of Adelaide, Adelaide, SA 5005, Australia; volker.hessel@adelaide.edu.au

**Keywords:** gold nanoparticels, activated carbon fibers, batch reactor, microreactor system

## Abstract

The process of noble metals ions recovery and the removal small fraction of nanoparticles from waste solution is an urgent topic not only from the economic but also ecology point of view. In this paper, the use of activated carbon fibers (ACF) as a “trap” for gold nanoparticles obtained by a chemical reduction method is described. The synthesized nanoparticles were stabilized either electrostatically or electrosterically and then deposited on carbon fibers or activated carbon fibers. Moreover, the deposition of metal on fibers was carried out in a batch reactor and a microreactor system. It is shown, that process carried out in the microreactor system is more efficient (95%) as compared to the batch reactor and allows for effective gold nanoparticles removal from the solution. Moreover, for similar conditions, the adsorption time of the AuNPs on ACF is shortened from 11 days for the process carried out in the batch reactor to 2.5 min in the microreactor system.

## 1. Introduction

Nanomaterials [1] are currently one of the most dynamically developing fields of science [2]. This is mainly due to their properties, which are dependent on their size. Precious metal nanoparticles are particularly popular due to their unique optical properties (e.g., silver [3], gold nanoparticles), catalytic properties (platinum [4], palladium [5]), and their susceptibility to surface modification by adding a reagent, which is required, for example, for medical purposes [6]. This makes nanoparticles useful in almost every field from engineering [7], energy [8], catalysis [9,10] to pharmacy and medicine [6,11]. However, each of these areas generates dangerous nano-waste [12,13,14]. This type of waste is difficult to monitor, due to its low concentration and “invisible” size. Thus, removal and recovery/recycling [15] is an important aspect of environmental protection.

There is little information in the literature on the removability of nanoparticles from waste solutions. Yun et al. [16] report that gold nanoparticles can be recovered from microbial biomass like marine bacterium Jeotgalibacillus using such compounds as sodium citrate and sodium dodecyl sulfate. Morita-Imura et al. [17] used for this purpose a pH-responsive self-assembly of zwitterionic amphiphile. Eastoe et al. [18] recovered gold nanoparticles using pH-induced heteroaggregation of mixed positive/negative aqueous microgels. The mentioned methods lead to gold aggregate formation and mostly require introducing additional compounds to the solution containing gold nanoparticles, which often is not ambient for the environment. It also appears that the practical application of these methods in the industry to remove nanoparticles from post-production waste may be problematic due to possible difficulties in the separation of aggregate gold.

In this context, the main aim of this work was to develop a new method of removing noble metal nanoparticles, including gold from waste solutions. For this purpose, we used commercially available carbon fibers, and their activated form, for AuNPs removal from synthetic solution. The carbon fibers were selected for their high chemical resistance and easy separation from the solution. In addition, gold deposited on the carbon surface can be recovered by hydrometallurgical methods such as the leaching process used in the recycling sector [19].

Moreover, the process of AuNPs adsorption was carried out in a batch reactor and in the microreactor system to show the possibility of enhancing the process of nanoparticles removal. We chose these reactors in order to compare the efficiency of the proposed process of nanowaste utilization and to show the possibilities of microreactor application to such purposes. It is known, that microreactors have great advantages [20] compared to a batch reactor, which are related to its miniaturization, that is, high surface to volume ratio, better temperature control, high operation temperature even for aqueous solution, better molecular diffusion, the possibility of separating each reaction product during the course of the reaction [21]. Due to these reasons, the microfluidic platform has found applications in the process of metal nanoparticles synthesis [22,23], nanoparticles with core-shell structure [24], particles synthesis in microdroplets [3,25], in process of metals extraction [26,27], even in the process of magnetic particles separation [28].

## 2. Materials and Methods

### 2.1. Chemicals

#### 2.1.1. Precursor of Gold Nanoparticles

As a metal precursor, the stock solution of 0.1 M tetrachloroauric acid was used, whose procedure of synthesis was described in our previous paper [29]. In order to prepare the solution for Au(III) ion adsorption and colloidal gold synthesis, the proper volume of gold(III) stock solution was diluted in deionized water or in 0.1 M HCl (p.a., Avantor Performance Materials Poland S.A.). Water, as a solvent, was used for two reasons. First, it does not increase the ionic strength of the solution (much lower than 0.01 M), which is a prerequisite for the operation of electrosteric stabilization. Second, this is the most frequently used solvent for metal nanoparticles synthesis due to its low price.

#### 2.1.2. Reductant and Electrostatic Stabilizer 

As a reductant and an electrostatic stabilizer, L-ascorbic acid (H_2_Asc, p.a., Avantor Performance Materials Poland S.A.) was used. For this purpose, a proper amount of L-ascorbic acid was dissolved in deionized water (stock solution 0.1 M). In experiments, a stock solution was next dissolved in order to obtain proper reductant concentration. It should be noted that the stock solution of reductant should not be stored longer than 8 h due to its oxidation with the oxygen from the air. For this reason, in all experiments, metal precursor and reductant were freshly prepared.

#### 2.1.3. Polymer

In order to obtain steric stabilization of colloidal gold, polyvinyl alcohol (PVA, 67,000 Da, Sigma-Aldrich, Taufkirchen, Germany) was used. An aqueous solution of the stabilizer was prepared in such a way, that 10 g of PVA powder was diluted in 100 mL of deionized water under ultrasound conditions and at temperature 40 °C. The solution thus obtained, after appropriate dilution, is ready for use. This solution can be stored for many months.

#### 2.1.4. Carbon Fibers as “a Trap” for Metal

In order to deposit gold particles, the commercially available carbon mate was used. In experiments, carbon fibers were used as obtained from the company and also after their surface activation was described in details in our previous work [30].

### 2.2. Synthesis and Deposition of Gold Nanoparticles

#### 2.2.1. Synthesis in the Batch Reactor

In order to perform gold nanoparticle synthesis, as well as deposition, the metal precursor was mixed (see, Table 1) with reductant in volume ratio 1:1 and in the presence of carbon fibers or activated carbon fibers in the batch with a volume of 25 mL (glass vessel, Duran, Mainz, Germany). Next, solutions were shaken together extensively for 2 min. After this time, the solutions were left in a thermostatic bath (20 °C) until reagents reacted.

#### 2.2.2. Synthesis in the Microreactor System 

**Figure 1:**
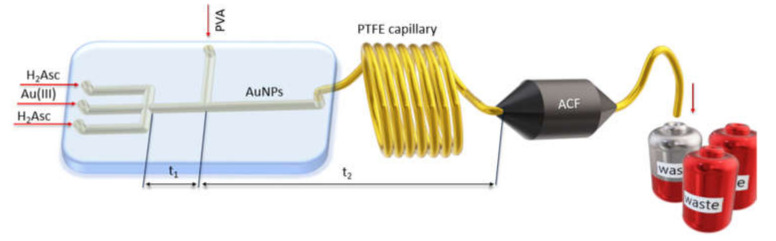
Scheme presented system for continuous particles synthesis and their deposition on the activated carbon surface, where t_1_—time for reduction (Au(III) to Au(I) ions); t_2_—time for nucleation and autocatalytic growth.

Gold nanoparticles were continuously synthesized in a microreactor system (glass chip with a diameter of the channel equal to 100 μm, Dolomite, Roystone, UK) connected with syringe pumps (Syrris, Royston, UK) and Teflon (PTFE) capillary (see, Figure 1). In the first step, streams of reductant (flow rate 1 mL/min, each) were mixed with gold(III) precursor (flow rate: 2 mL/min). Optionally, in the second step, the reacting compounds were mixed with a stream of polymer (flow rate: 1 mL/min). Next, formed colloidal gold was directed to the capillary ended with a filter containing ACF. After passing the filter the solution was directed to the waste.

### 2.3. Analysis Methods

#### 2.3.1. Spectra Analysis

Spectrophotometer UV-Vis UV–2501 PC (Shimadzu, Tokyo, Japan), working in the wavelength range 190–900 nm, was used to register the kinetics of the process and to analyze absorption spectra, respectively. The sample from batch synthesis were analyzed spectrophotometrically for several days (7–17). For this purpose, about 3 mL of the solution was pipetted and placed in a quartz cuvette with an optical path length of 1 cm (water was used as the reference solution). After analysis, the solution was returned to the reacting mixture. In the case of the synthesis of gold nanoparticles in the microreactor, the samples containing the colloidal gold were analyzed spectrophotometrically (absorption spectra studies) 10 min after collection of c.a. 2 mL of the solution. The sample of colloidal gold coming from experiments was examined twice (before and after passing through the filter with ACF by the solution containing colloidal gold).

#### 2.3.2. Dynamic Light Scattering (DLS) Analysis 

In order to measure the size and size distribution of obtained particles, Nanozetasizer Nano-S (Malvern Panalytical, Malvern, UK) was used. Directly after absorption spectra registration, the solution with colloidal gold was analyzed using the DLS method (size and size distribution).

#### 2.3.3. Scanning Electron Microscopy (SEM) Analysis

The microstructure observations were performed with the help of SEM (Hitachi SU-70, Hitachi, Tokyo, Japan). The samples for microscope analysis were performed after the colloidal gold synthesis and their deposition on activated carbon or carbon fibers. The fibers with metallic deposit were removed from the batch or from the filter in the case of the synthesis carried out in the microreactor system and dried at 50 °C over 4 h. After this time, the fibers were cooling down, and glued by Nafion^®^ to SEM sampler and again dried at room temperature for at least 12 h. The prepared sample was then analyzed using an SEM microscope.

#### 2.3.4. Fourier—Transform Infrared Spectroscopy

To study changes in the carbon surface induced during chemical modification samples of carbon and activated carbon fibers were analyzed using infra-red spectroscopy (Nicolet 380 FT–IR, Thermo Electron Corporation, Waltham, Massachusetts, USA). The spectra were registered in the range 500–4000 cm^−1^. For FT–IR analysis, 0.0012 g fibers were catted and gently mixed with dry KBr (0.2 g, FT–IR grade ≥ 99%, Sigma-Aldrich) in a mortar for one minute. Finally, the powder was pressed into a disc (200 bar) for about 1 min.

## 3. Results

### 3.1. Experimental Conditions

#### 3.1.1. Au(III) Ion Adsorption, Gold Nanoparticle Synthesis, and Deposition on Carbon/Activated Carbon Fibers Carried out in a Batch Reactor

For Au(III) ions, the process of colloidal gold synthesis and deposition on carbon fibers or activated carbon fibers was conducted in the batch reactor with a volume of 25 mL. For this purpose, different versions of experiments were performed whose details are gathered in Table 1.

#### 3.1.2. Gold Nanoparticles Synthesis and Deposition Carried out in a Microreactor System

The synthesis of colloidal gold and their deposition on activated carbon fibers (c.a. 0.01 g) was conducted under the following conditions: initial concentration of gold(III) ions in the range 0.05 mM to 0.30 mM and 0.9 mM for reductant, laboratory temperature (20 °C). Synthesis of colloidal gold was carried out with (samples AS4, AS6) and without polymer addition (samples AS3, AS5). The total flow rate of reagents in the flow system was set at values 4.0 (without PVA) or 5.0 mL/min (with PVA), based on kinetic data given in our previous study [31,32]. The scheme of the setup used for one-step AuNPs synthesis and deposition on ACF is shown in Figure 1.

As a result of the reaction of the gold(III) chloride complex with a reductant carried out in the flow, colloidal gold was obtained. The process of colloidal gold formation has three stages as shown in our previous work [33]. The gold nanoparticles were stabilized by L-ascorbic acid (electrostatic stabilizer) and PVA (steric stabilizer) (Figure 1). The flow rate of reagents was set as follows: the volume ratio of Au(III) ions to the reductant was 1:1 and the ratio of polymer to colloidal gold 1:4. The length of a microchannel (length of microreactor channel and length of PTFE capillary) was chosen in such a way, that the colloidal gold was formed before entering the filter containing the ACF (detailed explanation and calculation are given in SI, Section 1).

### 3.2. CF and ACF Characterization

**Figure 2:**
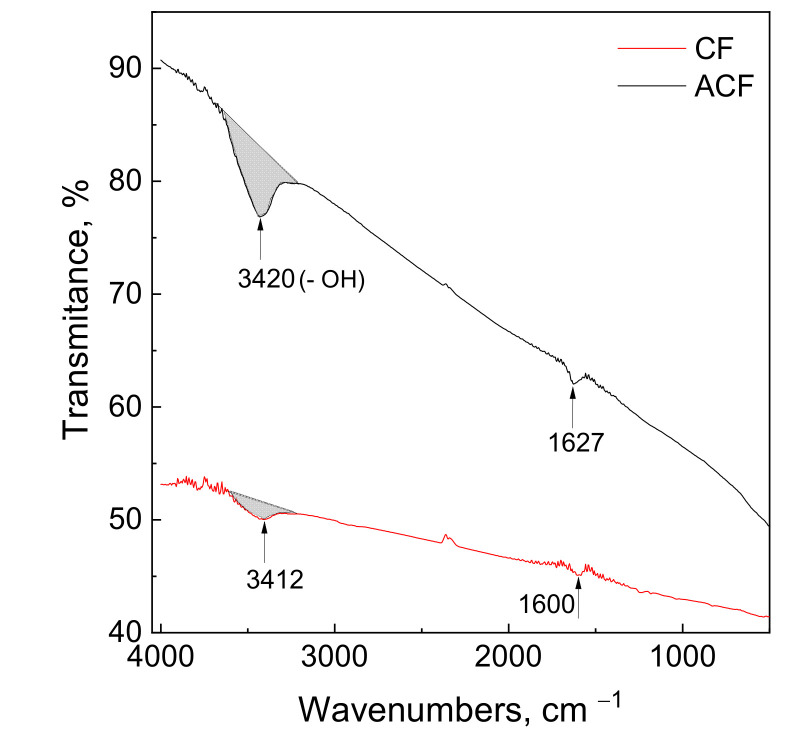
Fourier transform infrared spectroscopy spectra of carbon fibers before and after chemical treatment.

In Figure 2, FT–IR spectra of carbon and activated carbon fibers and the assignment of characteristic peaks are shown. It was observed that functional groups — OH, C = O, were found at 3200–3600 cm^−1^, 1500–1700 cm^−1^, respectively. These assignments are in good agreement with the literature [34].

In order to compare the amount of the surface functional group (-OH) present on CF and ACF, the same weight of fibers, as well as KBr, were used during FT–IR analysis. Thanks to that, semi quantities analyses were performed. For this purpose, we determined the area (grey area in Figure 2) of the peak of the broad signal between 3200 and 3600 cm^−1^ (v1.2.10, SpectraGryph software, Dr. Friedrich Menges Software—Entwicklung, Obersdorf, Germany). The amount of -OH functional groups is 3.6 times greater for ACF compared to CF. It was also observed (Figure 2) that broad signal between 1500–1700 cm^−1^ associated with a presence of the carbonyls group is also more intense in the case of ACF. Both groups (hydroxyl and carbonyls), according to literature are responsible for adsorption [35].

### 3.3. Process of Colloidal Gold Synthesis and Its Deposition on Carbon Fibers Carried out in the Batch Reactor

#### 3.3.1. Organoleptic Observation and Spectrophotometric Analysis of Colloidal Gold

**Figure 3:**
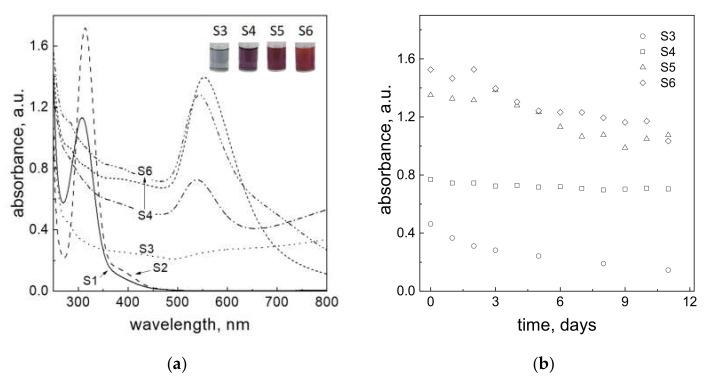
Spectra of solutions S1–S6 and the colour of colloidal gold obtained in the batch reactor (S3–S6) (**a**); Kinetic curves for process of colloidal gold adsorption on carbon fibers carried out in the batch reactor (**b**). Conditions: C_0, Au(III)_ = 0.3 mM, C_0, H2Asc_ = 0.6 mM, addition of PVA (samples S4, S6), amount of the carbon fibers 0.025 g/25 mL of the solution, T = 20 °C.

Depending on the conditions of performed experiments (see, Table 1), different colors (grey, violet, purple, and red, Figure 3a) of solutions after reagents mixing with carbon fibers were obtained. There is no image given for samples containing solutions S1 and S2 (only Au(III) ions) because no changes in color were observed after carbon fiber addition. The first analysis of obtained colloidal gold was made about 3 min after the reagents were mixed together. The obtained results are presented in Figure 3a.

In the case of experiments denoted as S1 and S2, the characteristic spectrum for Au(III) ions was registered at different solvents (see, Table 1). Taking into account the hydrolysis progress of Au(III) ions at different pH [32], in fact, in the solutions exist one or two forms. At pH = 1 (hydrochloric acid as a medium) there exists one form, that is, [AuCl_4_]^−^ whereas at pH values in the range 2–4 [32], there coexist two forms, that is, [AuCl_4_]^−^, [AuCl_3_(H_2_O)]. These forms have characteristic spectra with proper absorbance intensity and peak location (see Figure 3a). Thus, the characteristic peak for solution S1 is located at 308 nm, whereas for S2 at 314 nm, which is in accordance with the literature. In the case of solutions S3–S6, colloidal gold was obtained after reductant addition to the metal precursor. Registered spectra are shown in Figure 3a. The maximum of the peak was localized at a wavelength (λ_max_) about 540 nm (S4, S5) and at 650 nm (S6). It is worth noting, that only the spectrum in the visible wavelength range obtained for S5 is symmetrical, which suggests, that obtained AuNPs are uniform in morphology. Next, the process of Au(III) ion adsorption and gold nanoparticle deposition on carbon fibers in the batch reactor was monitored for up to 17 days. For this purpose, the change of the absorbance (according to Lambert–Beer Law A∝ AuNPs concentration) at λ_max_ was observed and based on that, the kinetic curves were determined (Figure 3b). For solutions S1 and S2, the kinetic curves (SI, Figure S1) were registered at 308 and 314 nm, respectively, and the absorbance corresponds to the change of Au(III) ions concentration. Generally, in the considered time, only a small change of absorbance values was observed. It suggests, that process of metal ion adsorption practically does not take place. In Figure 3b, the kinetic curves were registered at λ_max_ for solutions S3–S6. The small change in absorbance value suggests that process of gold nanoparticle deposition on carbon fibers is very slow. In addition, similar solutions to S5 and S6 were prepared without the addition of carbon fibers (samples S7 and S8, respectively). The obtained kinetic curves for these solutions are shown in SI, Figure S2 and show only small changes between them. These results also suggest, that colloidal gold itself changes with time and this observation probably corresponds to the process of AuNPs aggregation.

#### 3.3.2. SEM Analysis

After 17 days, the fibers were filtered, dried, and analyzed using SEM. Obtained results are shown in SI, Figures S3 and S4. For sample S1, a small amount of reduced gold was observed (SI, Figure S3a–c), whereas, for sample S2, there was no metallic gold on the carbon surface (SI, Figure S3d–e). As can be expected, the amount of AuNPs on carbon fibers was low or none (SI, Figures S3f,g and S4 ). However, some aggregates of metallic gold were also found (SI, Figure S4d). The obtained results show that the process of AuNPs synthesis and deposition in the batch reactor is ineffective, which could be expected. It is well known from the literature, that the adsorptive properties of carbon fibers are strongly dependent on the active surface of the adsorbent [36]. The surface area of carbon fiber can be increased via chemical oxidation [37] or thermal treatment [38]. Thus, in the next steps, the process of colloidal gold deposition on carbon fibers was carried out using ACF.

### 3.4. Process of Colloidal Gold Synthesis and Their Deposition on Activated Carbon Fibers Carried out in the Batch Reactor

#### 3.4.1. Organoleptic Observation and Spectral Analysis of Colloidal Gold

Depending on the variable of performed experiments (see, Table 1), the solutions (AS3–AS6) had a red color after mixing with activated carbon fibers. For solutions AS1 and AS2, no changes in the yellow color were observed and it was related to Au(III) ions.

**Figure 4:**
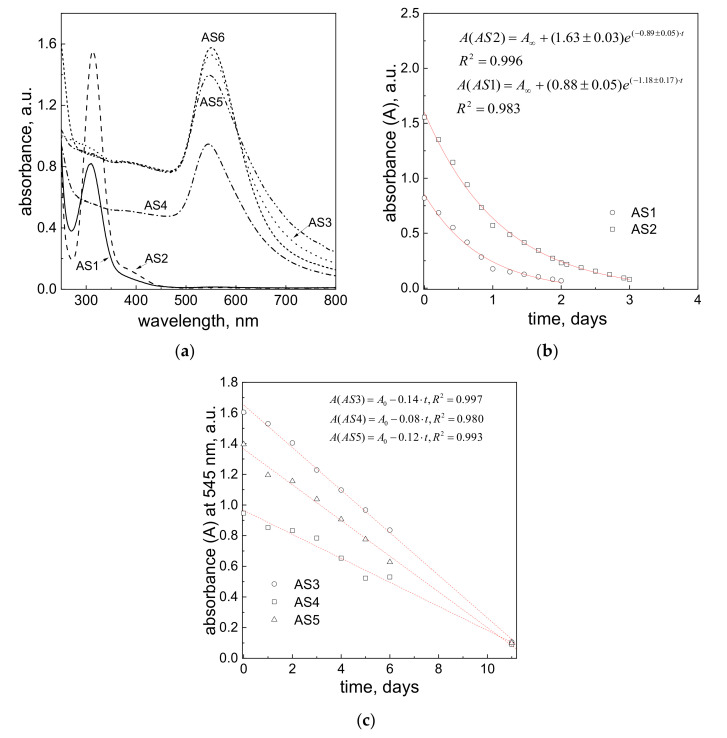
UV-Vis spectra of solutions: AS1–AS6 (**a**); Kinetic curves for process of adsorption of Au(III) ions at different media (samples AS1 and AS2) on activated carbon fibers carried out in the batch reactor (**b**); Kinetic curves for process of colloidal gold adsorption on activated carbon fibers carried out in the batch reactor (samples AS3–AS5) (**c**). Conditions: C_0, Au(III)_ = 0.3 mM, C_0, H2Asc_ = 0.6 mM, addition of PVA (sample AS4), amount of the activated carbon fibers 0.025 g/25 mL of the solution, T = 20 °C.

Analogously to the previous procedure, the first analysis of Au(III) ions at different media and synthesized colloidal gold were analyzed after about 3 min after the reagents were mixed with activated carbon fibers. The obtained results were presented in Figure 4a.

Samples AS1 and AS2 (see, Table 1) have, similarly to the S1, S2 spectra, characteristic absorbance bands with maxima at 308 nm (AS1) and 314 nm (AS2), respectively (see Figure 4a). However, the intensity of the spectra is a little bit lower compared to samples with carbon fibers (S1, S2, Figure 3a). This observation suggests, that Au(III) ion adsorb much faster on the ACF surface than on CF. These results are similar to the process of gold ion adsorption on different activated carbon surfaces like Norit ROX 0.8 [39], Calgon GRC-22 [40]. For samples denoted as AS3–AS6, the characteristic spectra were registered with the characteristic maximum located at 545 nm (AS3–AS5, Figure 4c) and 550 nm (AS6, SI, Figure S5), confirming the presence of colloidal gold. Moreover, absorption spectra obtained for AS3 and AS6 are symmetrical suggesting that obtained AuNPs are uniform in morphology. Next, the process of Au(III) ions and gold nanoparticle adsorption on ACF was monitored for 72 h (until the process was finished), whereas the process of AuNP deposition on ACF was analyzed for 11 days. Registered kinetic curves are shown in Figure 4b,c. For this purpose, the change in the UV-Vis spectrum was observed at a value of λ_max_ and based on that, the kinetic curves were determined (Figure 4b,c). For solutions AS1 and AS2, the kinetic curves were registered at 308 and 314 nm, respectively and according to the Lambert–Beer law they correspond to Au(III) ion concentration. Registered kinetic curves for samples AS1 and AS2 have an exponential character (see Figure 4b) and the adsorption process of ACF is much faster compared to samples S1 and S2 (carbon fibers). Moreover, the process of adsorption can be expressed by a kinetic equation in exponential form, which is in accordance with the literature [41,42]. Based on these equations, the values of the observed rate constants were determined, which were equal to 0.049 h^−1^ (AS1) and 0.037 h^−1^ (AS2). The process of adsorption was also carried out for colloidal gold. It can be seen, that process of adsorption runs much faster than in the case of deposition carried out on carbon fibers, and the process of sorption takes 11 days (except for the sample AS6, shown in SI, Figure S5). Registered at the wavelength of 540 nm, kinetic curves have a linear character (Figure 4c) and this suggests that the process of gold nanoparticle deposition on activated carbon fibers is in zero order. The difference in the sorption process mechanism is important. Typically, for the adsorption of Au(III) ions at the surface of activated carbon or activated carbon fibers, first-order kinetic curves are observed. Most often, mechanism studies show that the process is diffusion-controlled or mixed control. Only a few studies in the literature on the sorption of such large objects as nanoparticles on the surface of carbon structures have been reported. One would expect that such a process is purely diffusion-controlled, while the shape of the kinetic curve indicates that the mechanism is not obvious. It should be taken into account that the surface of the carbon fibers after the activation process is covered with numerous functional groups. This leads to a situation where two electrically charged surfaces interact with each other. In the case of nanoparticles, the charge is evenly distributed over the surface, in the case of carbon fibers and activated carbons, there is no such certainty.

#### 3.4.2. SEM Analysis

**Figure 5:**
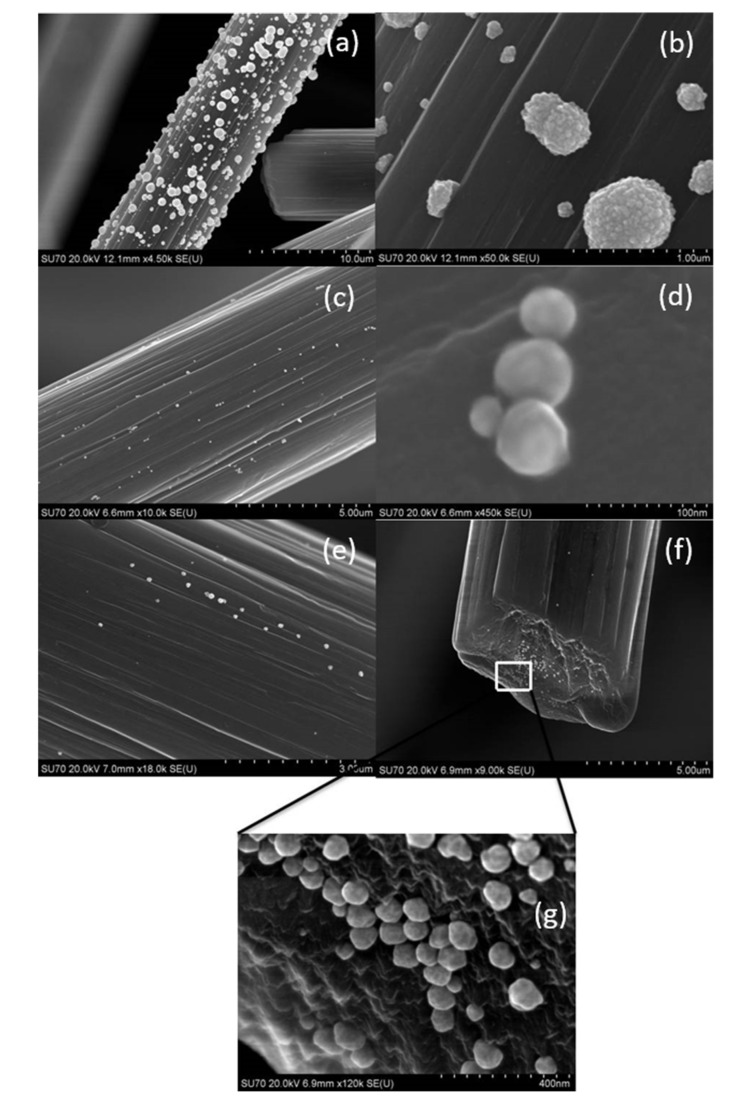
SEM analysis of activated carbon fibers covered by colloidal gold obtained during adsorption process carried out in the batch reactor: AS4 (**a**,**b**); AS5 (**c**,**d**); AS6 (**e**–**g**). Conditions: C_0, Au(III)_ = 0.3 mM, C_0, H2Asc_ = 0.6 mM, addition of PVA (samples AS4, AS6), amount of the activated carbon fibers 0.025 g/ 25 mL of the solution, T = 20 °C.

After 72 h or 11 days, the fibers were filtered, dried, and analyzed using SEM. Obtained results are shown in SI, Figure S6 (samples AS1–AS3), and Figure 4 and Figure 5 (samples AS4–AS6).

In the case of solutions AS1 and AS2, after the fast adsorption of Au(III) ions on activated carbon surface (Figure 4b), the process of metal ions reduction takes place and consequently slow nucleation and growth, which favors the formation of large crystallites (particles with size even 10 µm, SI, Figure S6a–e), is observed. As it can be expected, the amount of AuNP deposited on activated carbon fibers was also higher than in the case of gold deposited on CF (SI, Figure S6f,g and Figure 5), especially for sample AS4 (Figure 5a,b) compared to sample S4 (SI, Figure S4a,b). SEM analysis shows also a small number of gold particles with diameter c.a. 100 nm deposited on ACF (Figure 4 and Figure 5e–g).

### 3.5. Process of Colloidal Gold Synthesis and Their Deposition on Activated Carbon Fibers Carried out in the Microreactor System

Taking into account the results obtained from experiments carried out in the batch reactor, the activated carbon fibers as a “metal trap” were used for further studies. Moreover, in order to enhance the process of nanoparticle removal from the solution, a microreactor system was used. Here also, a different reagent concentration was applied in order to check how the size of synthesized nanoparticles affects the efficiency of the adsorption process on ACF. It is known from the literature, that in the case of ascorbic acid a higher reductant concentration compared to Au(III) ions leads to gold particles uncontrolled growth [33].

#### 3.5.1. Organoleptic Observation and Spectra Analysis of Colloidal Gold Synthesized in Microreactor System

**Figure 6:**
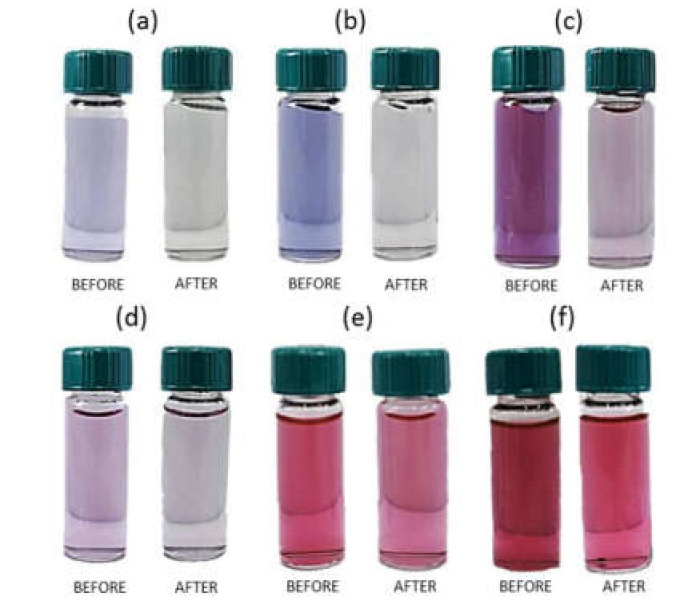
Colloidal gold obtained during reduction reaction of Au(III) ions with reductant. Experimental conditions: C_0, Au(III)_ = 0.05 mM, C_0, H2Asc_ = 0.9 mM (**a**); C_0, Au(III)_ = 0.15 mM, C_0, H2Asc_ = 0.9 mM (**b**); C_0, Au(III)_ = 0.3 mM, C_0, H2Asc_ = 0.9 mM (**c**), all colloids are without PVA. C_0, Au(III)_ = 0.05 mM, C_0, H2Asc_ = 0.9 mM (**d**); C_0, Au(III)_ = 0.15 mM, C_0, H2Asc_ = 0.9 mM (**e**); C_0, Au(III)_ = 0.3 mM, C_0, H2Asc_ = 0.9 mM (**f**), all colloids are with PVA.

Depending on the type of stabilization (electrostatic realized by ascorbic acid and electrosteric achieved by the presence of ascorbic acid and PVA), initial concentrations of the metal precursor and reductant, the colloidal gold with different colors: blue (Figure 6a,b), purple (Figure 6c), pink (Figure 6d), and red (Figure 6e,f), was obtained. The changing of the colors of the colloidal gold from blue to red is associated with a change in size (see, Section 3.5.2), shape as well as the polydispersity of the nanoparticles. During the process of Au nanoparticle deposition on the ACF, discoloration of the suspension (Figure 6a,b) was observed.

**Figure 7:**
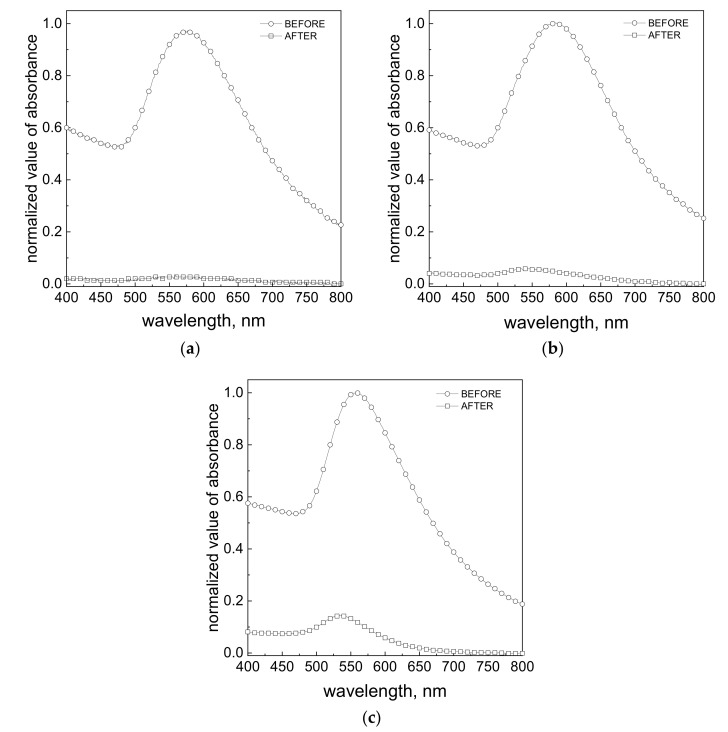
Absorption spectra of colloidal gold obtained in a result of reduction reaction of Au(III) ions with reductant. Experimental conditions: C_0, Au(III)_ = 0.05 mM, C_0, H2Asc_ = 0.9 mM (**a**); C_0, Au(III)_ = 0.15 mM, C_0, H2Asc_ = 0.9 mM (**b**); C_0, Au(III)_ = 0.3 mM, C C_0, H2Asc_ = 0.9 mM (**c**), all colloids are without PVA.

The obtained results shown in Figure 6 suggest that in each sample of colloidal gold, particles were sorbed on the ACF surface with different efficiency. Additional confirmation was achieved by registered spectra in the visible wavelength range. For this purpose, samples with colloidal gold were collected before and after the process of particle deposition on ACF. In each of the variants, the particle deposition process was associated with a clear decrease of the maximum absorbance values (Figure 7, SI, Figure S7). However, in the case where electrosteric stabilization was used, the absorbance value reduced to *c.a.* 50% (SI, Figure S7a), 15% (SI, Figure S7b), and 35% (SI, Figure S7c). This behavior suggests that the synthesized particles are less adsorbed on the ACF surface. Electrostatically stabilized particles are larger than those stabilized by a polymer and were much easier adsorbed on ACF. Moreover, ascorbic acid stabilized nanoparticles are more likely to agglomerate, which again improves the adsorption process.

Based on the obtained absorption spectra (see Figure 7), the wavelength values at which absorbance reaches its maximum were determined. The obtained results are summarized here (see SI, Table S1). The wavelength (λ_max_) for the colloids after flowing through the ACF shifts to the left in the case where electrostatic stabilization was applied. However, a similar relationship was not observed in the case of polymer-stabilized colloids. Such a wavelength shift may indicate that larger particles have deposited on the carbon support (better adsorptive properties), while smaller ones did not. Considering the fact that for this group of colloids (electrostatically stabilized) almost complete disappearance of the absorption band was observed (Figure 7a), it can be concluded that the adsorption process efficiency is between 85% (Figure 7c) and 95% (Figure 7a). No change in the position of the maxima of absorbance band was observed in the case of steric stabilized AuNPs. In addition, in the case of electrostatic stabilization, larger λ_max_ shifts toward shorter wavelengths were observed.

#### 3.5.2. DLS Analysis of Colloidal Gold Synthesized in the Microreactor

The results obtained from the dynamic light scattering method allowed the determination of the size of synthesized particles (SI, Table S1). The value of the hydrodynamic radius is described by the Stokes–Einstein diffusion equation:

(1) $$R_{h}=\frac{kT}{6\pi \eta D}$$

where: *R_h_*—hydrodynamic radius, *k*—Boltzmann factor, *T*—temperature, *η*—solvent viscosity, *D*—diffusion coefficient.

A hydrodynamic radius means that the determined radius also contains a layer of fluid adhering directly to the particle. Comparable radius values obtained in both stabilization methods do not mean that the particles are similar in size but indicate that the hydrodynamic layer is of a different thickness. In the case of polymeric stabilization, it is definitely thicker due to the nature of the interaction between the steric polymer and the particle, that is, adsorption of the stabilizer on the surface of the nanoparticle. It was also observed that the size of the hydrodynamic radius is about 10 nm smaller in the case of colloid obtained after passing through ACF and it suggests, that larger particles adsorb more easily.

#### 3.5.3. SEM Analysis

**Figure 8:**
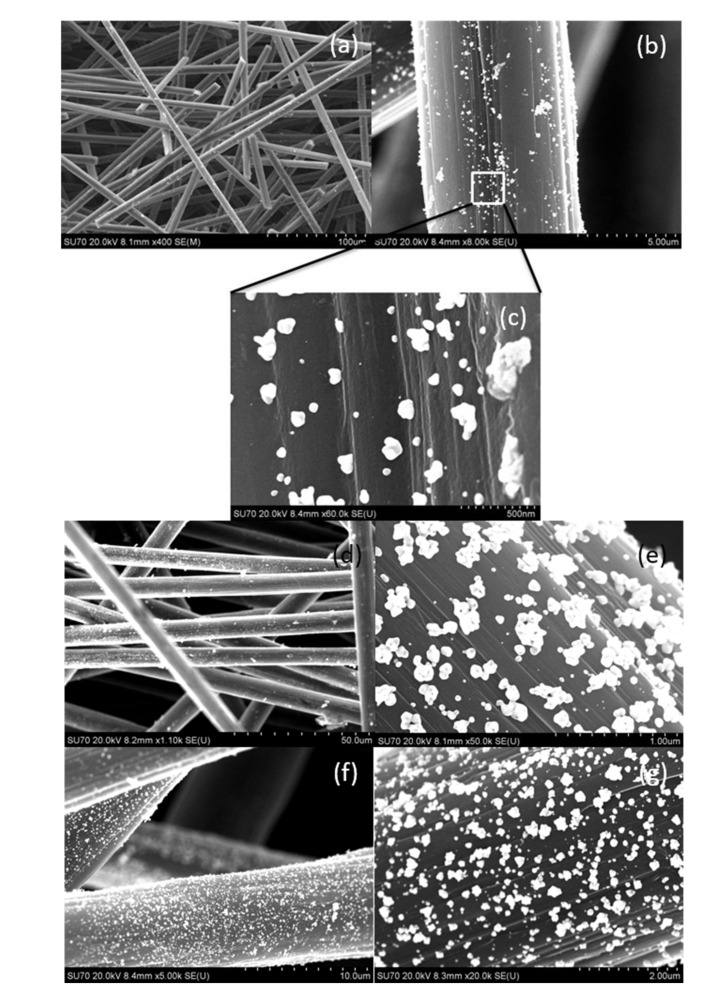
Photos taken with a scanning microscope showing the appearance of activated carbon fibers (**a**) and enlargement of the gold deposition (**b**,**c**). The synthesis conditions of Au/ACF: C_0, Au(III)_ = 0.05 mM, C_0, H2Asc_ = 0.9 mM, (**a**–**c**); the synthesis conditions of Au/ACF: C_0, Au(III)_ = 0.15 mM, C_0, H2Asc_ = 0.9 mM (**d**,**e**); the synthesis conditions of Au/ACF: C_0, Au(III)_ = = 0.3 mM, C_0, H2Asc_ = 0.9 mM (**f**,**g**). All experiments are without the addition of PVA to the system.

The final stage of the analysis was the observation of appropriately prepared samples under the scanning microscope. The obtained results showed that Au nanoparticles were deposited on the ACF surface in each case (Figure 8 and SI, Figure S8). However, different coverage was observed depending on experimental conditions. In the case of electrostatic stabilization, the size of deposited nanoparticles varied from 50 to 250 nm (Figure 8). A higher initial concentration of gold(III) ions used for AuNPs synthesis leads to the higher coverage of the ACF surface (Figure 8). The particle size differs significantly from the values obtained by the DLS method. However, the results from DLS relate to particles size analysis in the aqueous phase, while, the SEM analysis shows particles adsorbed on the carbon surface. It is clear that on the carbon surface, the deposited particles might further grow (i.e., Ostwald ripening) to form characteristic objects with sizes much larger than that determined in the aqueous phase. In the case of steric stabilization (addition of PVA), with the increase of the concentration of particles (increase in the initial concentration of Au (III) ions) a decrease in the size of deposited gold particles was observed (Figure S8). The size of the nanoparticles ranged from 20–100 nm. It can be seen that for higher concentrations of nanoparticles, a higher monodispersity was obtained.

## 4. Discussion

The process of AuNP adsorption was carried out in batch (using carbon and activated carbon fibers) and microreactor systems (only activated carbon fibers). Synthesized particles were stabilized electrostatically (using ascorbic acid) and electrosterically (realized by a mixture of both ascorbic acid and polymer). It was shown that carbon fibers without further chemical modification are ineffective. There were no observed changes both in the Au(III) ions and in the AuNPs concentration with time.

The application of ACF as the gold nanoparticles trap can be a promising method for Au(III) ion and particle removal from aqueous waste solutions. Moreover, it was shown, that the efficiency of the process of gold nanoparticle deposition on ACF can be enhanced in the microreactor system in which efficiency reached 95% for colloidal gold stabilized electrostatically. In the case of nanoparticles stabilized electrosterically, it seems that the process of adsorption is hampered. Here, a significant impact is due to the presence of the functional groups being on the ACF surface (among them: hydroxyl and carbonyls), which are the source of the surface charge. This charge may effectively interact with the surface charge of the AuNPs (electrosterical stabilization). The particle surface charge can be shielded by a polymer shell during steric stabilization. This causes the process of adsorption to be ineffective. It was also observed, that fraction of smaller particles (SI, Table S1) is difficult to deposit on the ACF surface, and they were detected in the waste stream. As said before, these particles were stabilized by a polymer, and thus it is difficult to clearly indicate whether small particles actually adsorb easier. It was also shown that during adsorption, AuNPs with sizes above 200 nm and larger aggregates can be deposited on activated carbon fibers. This result may be promising for the process of AuNP recovery from ACF. The recovery process may be realized by the combustion of the ACF process or simply by the dissolution of the deposit. Moreover, it was shown, that the application of a microreactor system allows for the acceleration of the process of AuNP adsorption and shortened process time from days (Figure 4c) to a few minutes (Figure 7), which is dependent on the total flow rate of reagents.

## 5. Conclusions

The process of Au(III) ion adsorption on activated carbon fibers carried out in the batch reactor is pseudo-first-order for which the values of observed rate constants were determined and are equal to 0.049 h^−1^ (AS1) and 0.037 h^−1^ (AS2). The process of AuNP adsorption on activated carbon fibers carried out in the batch reactor is zero-order and indicates a complex mechanism. The values of observed zero-order rate constants were established as: 0.0058 mol·dm^3^ h^−1^ (AS3), 0.0033 mol·dm^3^ h^−1^ (AS4), and 0.005 mol·dm^3^ h^−1^ (AS5). It was shown, that the application of microreactor enhances the process of AuNP deposition on ACF (efficiency reached 95% for colloidal gold stabilized electrostatically). Moreover, the time of the adsorption process carried out in the microreactor is much shorter as compared to the batch reactor. For similar conditions, this time is shortened from 11 days to 2.5 min (time needed to collect 10 mL of the sample, a total flow rate of reagents 4 mL/min) or 2 min for a total flow rate of 5 mL/min. The obtained results are promising for the process of removing gold nanoparticles from waste solutions, especially in the case of electrostatically stabilized particles. Particles deposited on ACF tend to form large aggregates and/or continue further growth, which leads to the accumulation of gold on the carbon surface facilitating its subsequent recovery.

## Figures and Tables

**Table 1:** Experimental conditions for the process of Au(III) ions and AuNP adsorption on carbon fibers (also activated) carried out in the batch reactor. Conditions: C_0, Au(III)_ = 0.3 mM, C_0, H2Asc_ = 0.6 mM, addition of PVA (mass ratio of Au(III) to polymer equals 1:10), amount of the carbon/ activated carbon fibers 0.025 g/25 mL of the solution, T = 20 °C.

Notation	Carbon Fibers	Type of Stabilization
S1	Carbon fibers without further modification	none ^1a^
S2		none ^1b^
S3		electrostatic ^2^
S4		electrosteric ^2^
S5		electrostatic ^3^
S6		electrosteric ^3^
AS1	Activated carbon fibers	none ^1a^
AS2		none ^1b^
AS3		electrostatic ^2^
AS4		electrosteric ^2^
AS5		electrostatic ^3^
AS6		electrosteric ^3^

The order of the individual components mixing: ^1a^ Only Au(III) ions (H_2_O as a solvent) mixed with carbon fibers. ^1b^ Only Au(III) ions (0.1 M HCl as a medium) mixed with carbon fibers. ^2^ Carbon fibers mixed first with Au(III) ions and then with a reductant. ^3^ Carbon fibers mixed with colloidal gold (first reagents, i.e., Au(III) with reductant were simultaneously mixed in the batch reactor).

## Data Availability

Additional results are contained in Supplementary Material.

## Supplementary Materials

The following are available online at https://www.mdpi.com/article/10.3390/ma14216598/s1, Figure S1. Kinetic curves for process of Au(III) ion adsorption on carbon fibers carried out in the batch reactor. S1—H_2_O as solvent; S2—0.1 M HCl as solvent. Conditions: C_0, Au(III)_ = 0.3 mM, T = 20 °C, Figure S2: Kinetic curves for process of colloidal gold adsorption on carbon fibers carried out in the batch reactor and behavior of AuNPs with time in the similar samples to S5 and S6 but without carbon fibers in the solution (S7,S8). Conditions: C_0, Au(III)_ = 0.3 mM, C_0, H2Asc_ = 0.6 mM, without PVA (S5, S7), addition of PVA (samples S6, S8), amount of the carbon fibers 0.025 g/25 mL of the solution, T = 20 °C, Figure S3. SEM analysis of carbon fibers covered by colloidal obtained during adsorption process carried out in the batch reactor. S1 (a–c); S2 (d–e); S3 (f–g). Conditions: C_0, Au(III)_ = 0.3 mM, C_0, H2Asc_ = 0.6 mM, amount of the carbon fibers 0.025 g/ 25 mL of the solution, T = 20 °C, Figure S4. SEM analysis of carbon fibers covered by colloidal gold obtained during adsorption process carried out in the batch reactor. S4 (a, b); S5 (c, d); S6 (e-f). Conditions: C_0, Au(III)_ = 0.3 mM, C_0, H2Asc_ = 0.6 mM, addition of PVA (samples S4, S6), amount of the carbon fibers 0.025 g/25 mL of the solution, T = 20 °C, Figure S5. Kinetic curve for process of colloidal gold adsorption on carbon fibers carried out in the batch reactor (sample AS6). Conditions: C_0, Au(III)_ = 0.3 mM, C_0, H2Asc_ = 0.6 mM, addition of PVA (sample AS6), amount of the activated carbon fibers 0.025 g/25 mL of the solution, T = 20 °C, Figure S6. SEM analysis of carbon fibers covered by colloidal obtained during adsorption process carried out in the batch reactor: AS1 (a–c); AS2 (d, e); AS3 (f, g). Conditions: C_0, Au(III)_ = 0.3 mM, C_0, H2Asc_ = 0.6 mM, amount of the activated carbon fibers 0.025 g/ 25 mL of the solution, T = 20 °C, Figure S7. Absorption spectra of colloidal gold obtained in results reduction reaction of Au(III) ions with L-ascorbic acid. Experimental conditions: C_0, Au(III)_ = 0.05 mM, C0, C_0, H2Asc_ = 0.9 mM (a); C_0, Au(III)_ = 0.15 mM, C_0, H2Asc_ = 0.9 mM (b); C_0, Au(III)_ = 0.3 mM, C_0, H2Asc_ = 0.9 mM (c), all samples contain PVA, Figure S8. SEM analysis of activated carbon fibers covered by colloidal obtained during adsorption process carried out in the microreactor: activated carbon fibers (a); C_0, Au(III)_ = 0.05 mM, C_0, H2Asc_ = 0.9 mM (b, c); C_0, Au(III)_ = 0.15 mM, C_0, H2Asc_ = 0.9 mM (d); C_0, Au(III)_ = 0.3 mM, C_0, H2Asc_ = 0.9 mM (e), Table S1: List of the absorbance (A_max_) and corresponding wavelength (λ_max_) values from spectrophotometric measurements and the hydrodynamic radius (R_H_) value obtained by the DLS method. Where R_HI_ and R_HN_ are respectively the hydrodynamic radius measured after intensity and after the number for the gold colloid BEFORE and AFTER the flow through ACF.
