# Supplementary material for: Batch Reactor vs. Microreactor System for Efficient AuNP Deposition on Activated Carbon Fibers
Source: Materials (Basel). 2021 Nov 2;14(21):6598. doi: 10.3390/ma14216598 (PMC8585125; doi:10.3390/ma14216598)
Supplement: Supplementary file 1 [file materials-14-06598-s001.zip › materials-1409273-supplementary.pdf]

materials

MDPI

Supplementary Materials

Batch Reactor vs. Microreactor System for Efficient AuNP Deposition on Actiavated Carbon Fibers

Magdalena Luty-Błocho [1,*], Marek Wojnicki [1], Tomasz Tokarski [2], Volker Hessel [3] and Krzysztof Fitzner [1]

[1] Faculty of Non-Ferrous Metals, AGH University of Science and Technology, Al. A. Mickiewicza 30, 30-059 Krakow, Poland; marekw@agh.edu.pl (M.W.); fitzner@agh.edu.pl (K.F.)
[2] Academic Centre for Materials and Nanotechnology, AGH University of Science and Technology, Al. A. Mickiewicza 30, 30-059 Kraków, Poland; tokarski@agh.edu.pl
[3] School of Chemical Engineering and Advanced Materials, University of Adelaide, Adelaide, SA 5005, Australia; volker.hessel@adelaide.edu.au
* Correspondence: mlb@agh.edu.pl

1. Parameters of the Microreactor System

The process of AuNPs synthesis in the system containing aqueous solutions of Au(III) chloride complex ions and ascorbic acid via chemical reduction method is three stages as was described in detail in our previous work [1,2]. In the first step Au(III) are reduced to Au(I) ions, in next Au(I) ions are reduced to Au(0) (nucleation) and in the last step the autocatalytic growth takes place. Each step can be described by kinetic equations and values of rate constants determined. However, depending on the initial conditions (reagents concentration, temperature, pH) not all processes can be measured and not all data (e.g. rate constants) determined. For our purposes, we established approximation microreactor system parameters, especially length of PTFE capillary, based on kinetic data related to first step in process of AuNPs formation [1] and related to nucleation and growth [2]. Based on these data, for similar conditions the half time was determined for the first step. Knowing that the reduction Au(III) to Au(I) ions is pseudo- first order, thus half time of this step can be described by equation:

$$t_{1/2} = \frac{ln2}{k_{1,obs}} \quad (1)$$

In our conditions (pH c.a. 3) it was not possible to determine the values of observed rate constant ($k_{1,obs}$), because the reaction reduction of Au(III) to Au(I) ions is too fast as was shown in the literature [1]. However, we made some estimations, and we used data i.e. the value of observed rate constant for lower concentration of ascorbic acid (i.e. 0.3 mM) for which observed rate constants equals 182.9 s^{-1}. For this data, the value of half time is equal 3.8 ms. In real conditions (concentration of ascorbic acid 0.9 mM), time (t_1) is less than 3.8 ms and for calculation we assumed that $t_1 = t_{1/2}$. Taking into account established flow rate of reagents (4.0 or 5.0 mL/min) we calculated the length of the capillary (the length of the microchannel due to small volume has been neglected), i.e. distance between point in which streams of reductant and metal ions are connected to the entrance a stream with polymer. The value of the length was denoted as L_1 and this value corresponds to time (t_1, scheme shown in Figure 1). Taking into account that flow rate is described as:

$$t_{1/2} = \frac{ln2}{k_{1,obs}} \quad (2)$$

where: F_R—flow rate, mL/min; V—volume, mL; t_1—time, s; D—capillary diameter, cm; L—capillary length, cm.

Knowing the value of flow rate, an inner capillary diameter (0.8 mm) and time t_1 we calculate the value of L_1 and it equals 3 cm. In next step, we approximate the length capillary L_2 (i.e. distance between entrance of polymer stream and filter with activated carbon fibers) before filter with activated carbon fibers. For this purpose we used time t_2 = 3 s which is needed to the process of AuNPs formation to be completed [2]. Based on this data the length of the capillary was established and equals 40 cm for flow rate 4.0 mL/min and 50 cm for 5.0 mL/min.

2. Process of Adsorption of Au(III) Ions and AuNPs on Carbon Surface Carried out in Batch Reactor

2.1. Adsorption of Au(III) Ions at Different Solvents on Carbon Surface

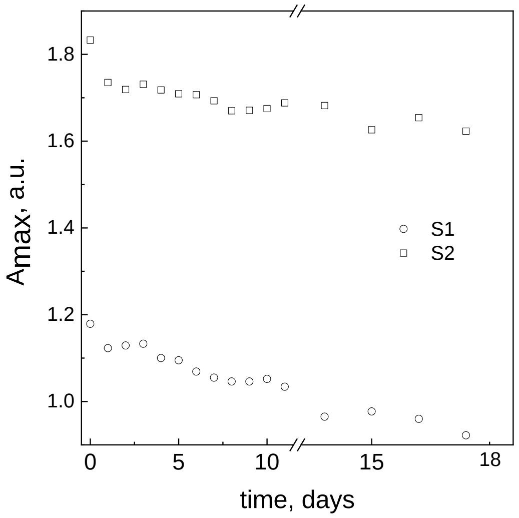

Figure S1. Kinetic curves for process of Au(III) ions adsorption on carbon fibers carried out in the batch reactor. S1—H_2O as solvent; S2—0.1 M HCl as solvent. Conditions: $C_{0, Au(III)}$ = 0.3 mM, T = 20 °C.

2.2. Registered Kinetic Curves for AuNPs in Selected Conditions.

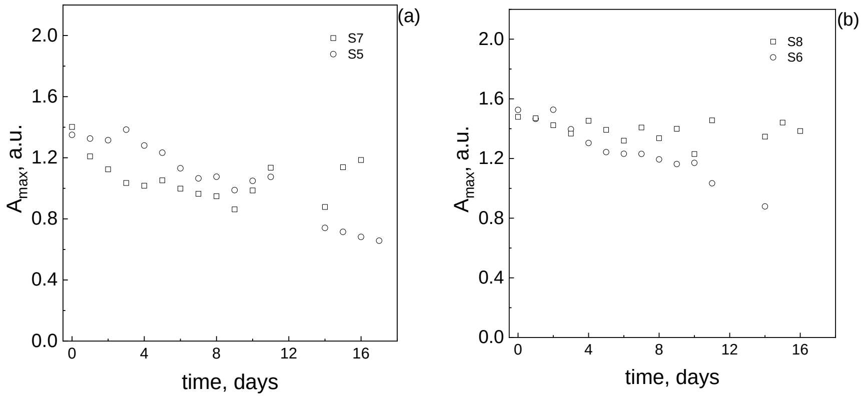

Figure S2. Kinetic curves for process of colloidal gold adsorption on carbon fibers carried out in the batch reactor and behavior of AuNPs with time in the similar samples to S5 and S6 but without carbon fibers in the solution (S7, S8). Conditions: $C_{0,\ Au(III)}$ = 0.3 mM, $C_{0,\ H2Asc}$ = 0.6 mM, without PVA (S5, S7), addition of PVA (samples S6, S8), amount of the carbon fibers 0.025 g/25 mL of the solution, T = 20 °C.

2.3. SEM Analysis for Process of Au(III) and AuNPs Adsorption on Carbon Fibers.

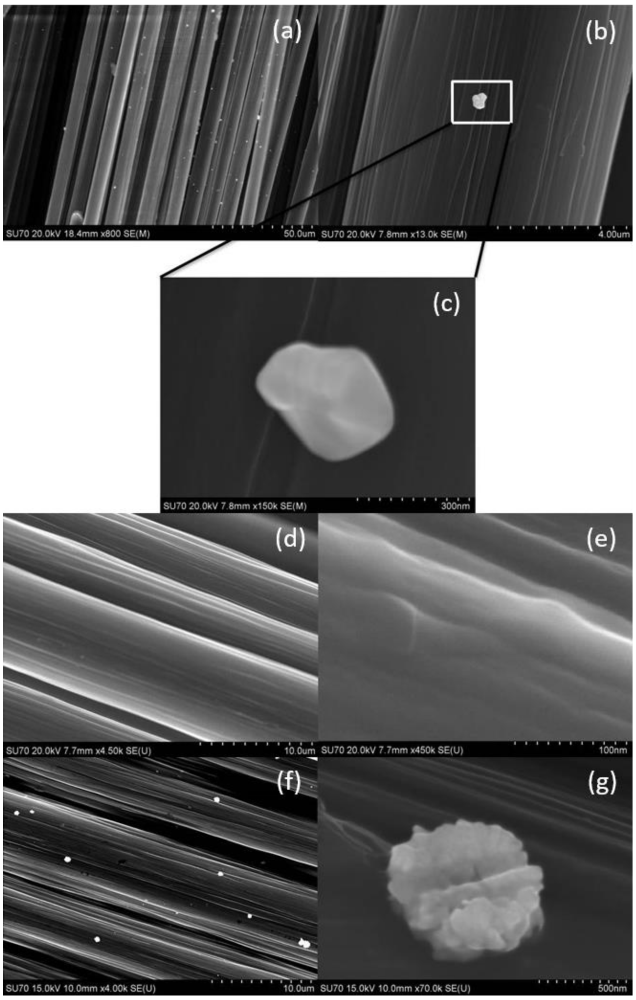

Figure S3. SEM analysis of carbon fibers covered by colloidal obtained during adsorption process carried out in the batch reactor. S1 (**a**–**c**); S2 (**d**–**e**); S3 (**f**–**g**). Conditions: $C_{0,\ Au(III)}$ = 0.3 mM, $C_{0,\ H2Asc}$ = 0.6 mM, amount of the carbon fibers 0.025 g/25 mL of the solution, T = 20 °C

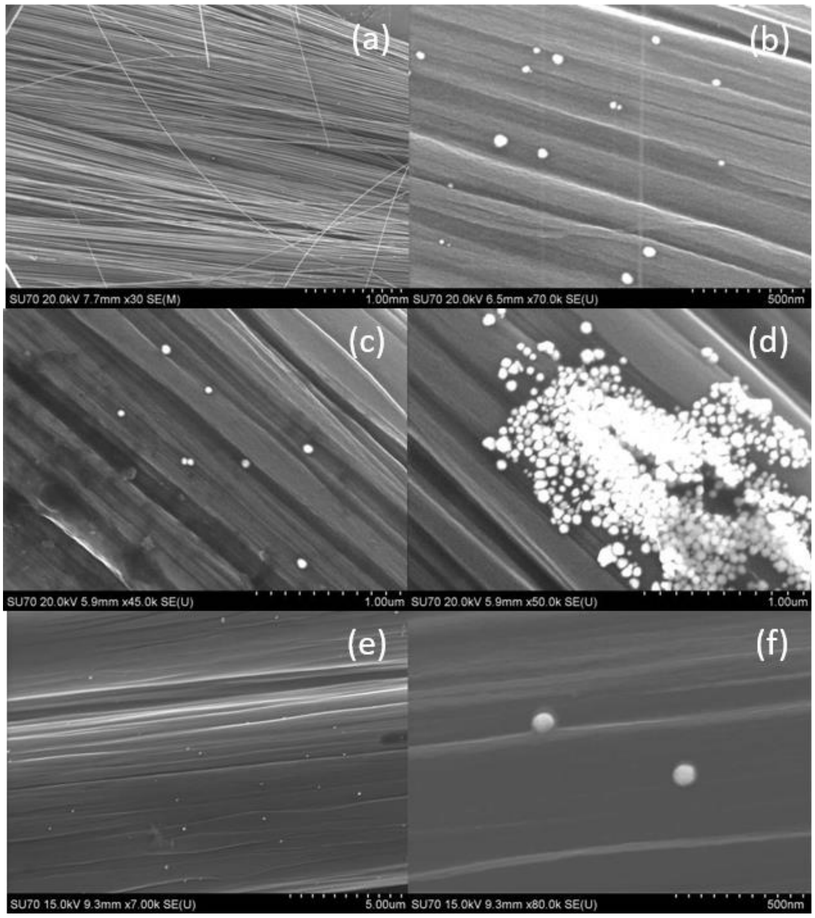

Figure S4. SEM analysis of carbon fibers covered by colloidal gold obtained during adsorption process carried out in the batch reactor. S4 (**a**,**b**); S5 (**c**,**d**); S6 (**e**–**f**). Conditions: $C_{0,\ Au(III)}$ = 0.3 mM, $C_{0,\ H2Asc}$ = 0.6 mM, addition of PVA (samples S4, S6), amount of the carbon fibers 0.025 g/25 mL of the solution, T = 20 °C.

3. Process of AuNPs Adsorption on Activated Carbon Surface Carried out in the Batch Reactor

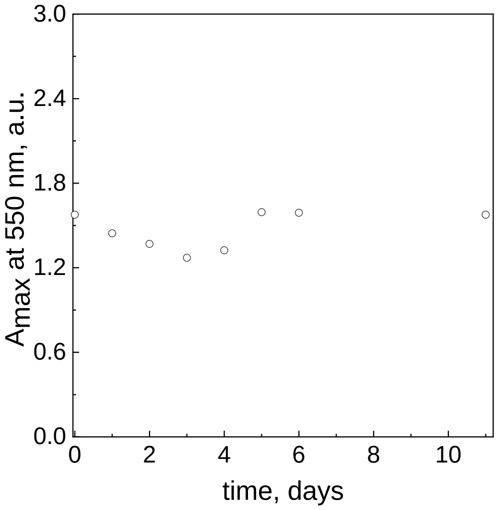

Figure S5. Kinetic curve for process of colloidal gold adsorption on carbon fibers carried out in the batch reactor (sample AS6). Conditions: $C_{0,\ Au(III)}$ = 0.3 mM, $C_{0,\ H2Asc}$ = 0.6 mM, addition of PVA (sample AS6), amount of the activated carbon fibers 0.025 g/25 mL of the solution, T = 20 °C.

3.1. SEM Analysis for Process Adsorption of Au(III) and AuNPs on Activated Carbon Fibers

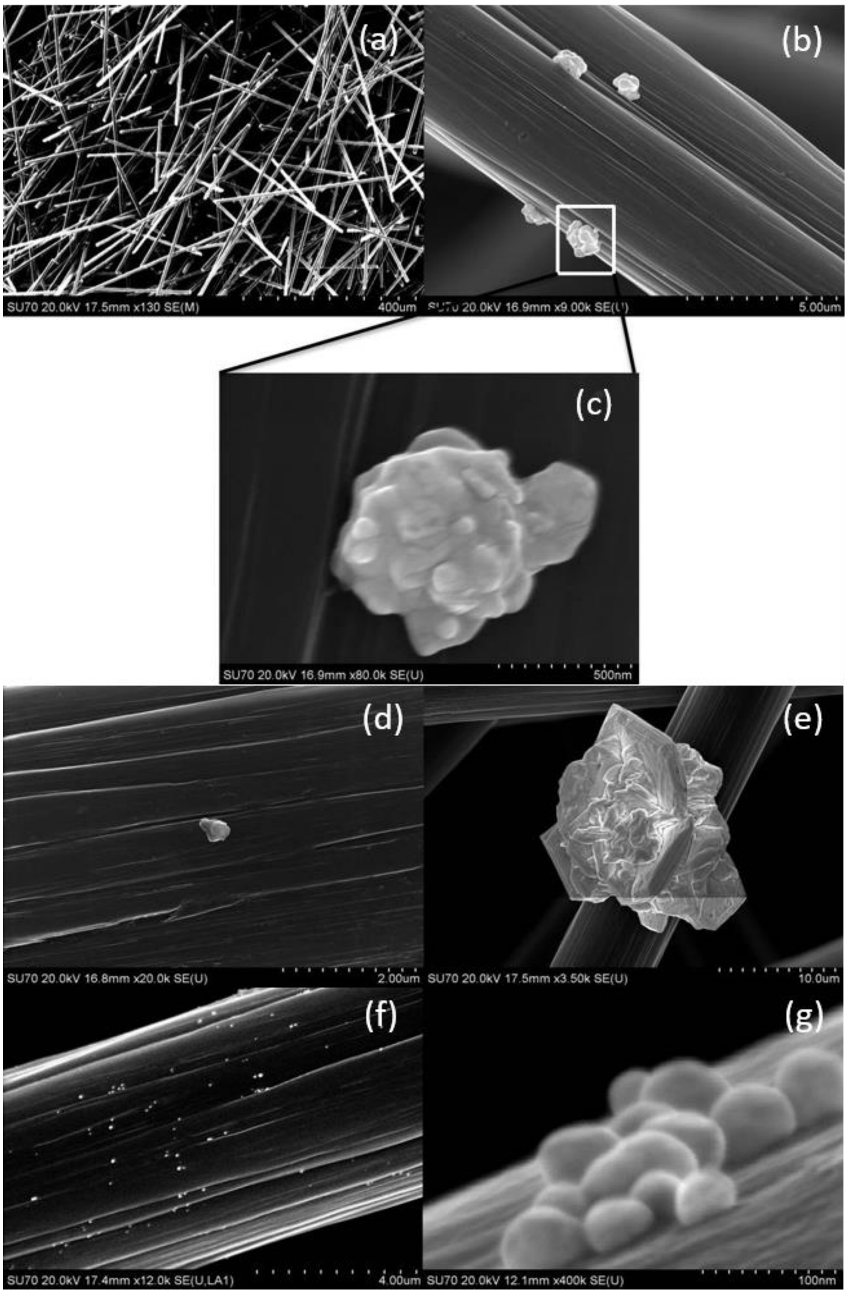

Figure S6. SEM analysis of carbon fibers covered by colloidal obtained during adsorption process carried out in the batch reactor: AS1 (**a**–**c**); AS2 (**d**,**e**); AS3 (**f**,**g**). Conditions: $C_{0,\ Au(III)}$ = 0.3 mM, $C_{0,\ H2Asc}$ = 0.6 mM, amount of the activated carbon fibers 0.025 g/25 mL of the solution, T = 20 °C.

4. Process of AuNPs Adsorption on Activated Carbon Surface Carried out in the Microreactor

4.1. Spectra Analysis

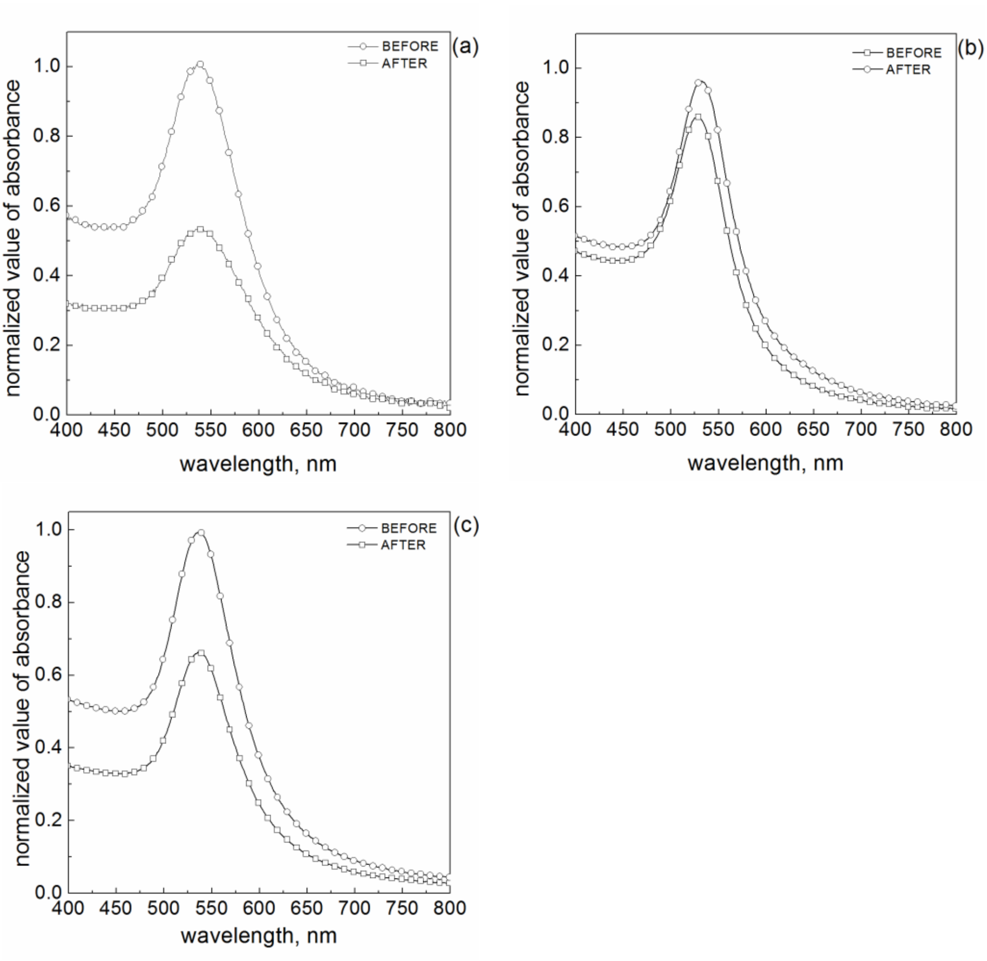

Figure S7. Absorption spectra of colloidal gold obtained in results reduction reaction of Au(III) ions with L-ascorbic acid. Experimental conditions: $C_{0,\ Au(III)}$ = 0.05 mM, $C_{0,\ H2Asc}$ = 0.9 mM (a); $C_{0,\ Au(III)}$ = 0.15 mM, $C_{0,\ H2Asc}$ = 0.9 mM (b); $C_{0,\ Au(III)}$ = 0.3 mM, $C_{0,\ H2Asc}$ = 0.9 mM (c), all samples contain PVA.

4.2. DLS Analysis

Table S1. List of the absorbance (A_{max}) and corresponding wavelength (λ_{max}) values from spectrophotometric measurements and the hydrodynamic radius (R_H) value obtained by the DLS method. Where R_{HI} and R_{HN} are respectively the hydrodynamic radius measured after intensity and after the number for the gold colloid BEFORE and AFTER the flow through ACF.

$C_{0,Au(III)}$, mM	polymer	λ_{max}, nm		A_{max}, a.u.		R_{HI}, nm		R_{HN}, nm	
		Before	After	Before	After	Before	After	Before	After
0.05	Without PVA	576	560	0.146	0.04	44.77 ± 17.40	33.55 ± 8.80	23.81 ± 7.57	24.43 ± 5.94
0.15		582	545	0.345	0.02	42.70 ± 15.32	31.43 ± 11.30	24.23 ± 7.49	17.69 ± 5.22
0.30		556	535	0.823	0.118	53.42 ± 17.69	33.51 ± 11.98	33.57 ± 10.30	19.01 ± 5.58
0.05	With PVA	540	540	0.180	0.08	46.64 ± 17.79	40.03 ± 15.09	25.33 ± 8.08	21.88 ± 6.73
0.15		533	532	0.540	0.528	52.68 ± 21.08	47.19 ± 18.91	27.62 ± 9.17	24.01 ± 7.90
0.30		537	537	0.796	0.530	60.49 ± 23.47	75.87 ± 29.32	33.48 ± 11.45	45.86 ± 16.28

4.3. SEM Analysis

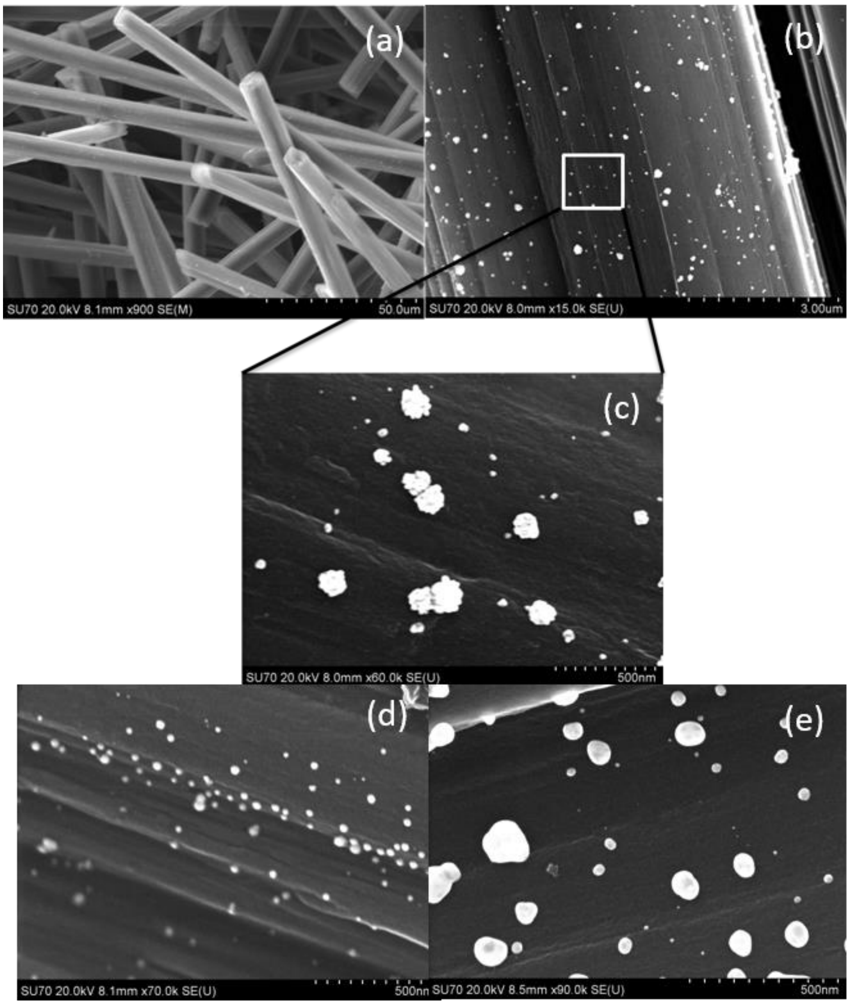

Figure S8. SEM analysis of activated carbon fibers covered by colloidal obtained during adsorption process carried out in the microreactor: activated carbon fibers (**a**); $C_{0,\ Au(III)}$ = 0.05 mM, $C_{0,\ H2Asc}$ = 0.9 mM (**b**,**c**); $C_{0,\ Au(III)}$ = 0.15 mM, $C_{0,\ H2Asc}$ = 0.9 mM (d); $C_{0,\ Au(III)}$ = 0.3 mM, $C_{0,\ H2Asc}$ = 0.9 mM (e).

Reference

1. Luty-Błocho, M.; Pacławski, K.; Wojnicki, M.; Fitzner, K. The kinetics of redox reaction of gold(III) chloride complex ions with l-ascorbic acid. *Inorganica Chim. Acta* **2013**, *395*, 189–196, doi:10.1016/j.ica.2012.10.031.
2. Luty-Błocho, M.; Wojnicki, M.; Fitzner, K. Gold Nanoparticles Formation via Au(III) Complex Ions Reduction with l -Ascorbic Acid. *Int. J. Chem. Kinet.* **2017**, *49*, 789–797, doi:10.1002/kin.21115.
